# High-dose cannabidiol for chronic neuropathic pain associated with spinal cord injury: a randomised clinical trial

**DOI:** 10.1016/j.eclinm.2026.103986

**Published:** 2026-05-28

**Authors:** Rebecca V. Robertson, Anastasia Suraev, Danielle McCartney, Allan Peng, Noemi Meylakh, Rebecca Gordon, Fernando A. Tinoco-Mendoza, Callum Morse, Leana Sattarov, Claire L. Boswell-Ruys, Kevin A. Keay, Elizabeth A. Cairns, Sachin Shetty, Iain S. McGregor, Luke A. Henderson

**Affiliations:** a School of Medical Sciences (Neuroscience), University of Sydney, Camperdown, New South Wales, 2006, Australia; b Brain and Mind Centre, University of Sydney, Camperdown, New South Wales, 2006, Australia; c Lambert Initiative for Cannabinoid Therapeutics, The University of Sydney, Sydney, New South Wales, Australia; d School of Psychology, Faculty of Science, The University of Sydney, Sydney, New South Wales, Australia; e School of Pharmacy, Faculty of Medicine and Health, The University of Sydney, Sydney, New South Wales, Australia; f Prince of Wales Hospital, Randwick, New South Wales, 2031, Australia; g Neuroscience Research Australia, Randwick, New South Wales, 2031, Australia; h University of New South Wales, Kensington, New South Wales, Australia

**Keywords:** Neuropathic pain, Cannabidiol, Pain treatment, Analgesia

## Abstract

**Background:**

Chronic neuropathic pain is common after spinal cord injury (SCI), yet current treatments have limited efficacy and significant side effects. Cannabidiol (CBD), a non-intoxicating component of cannabis, has demonstrated efficacy in preclinical neuropathic pain models. Here, we investigated the effect of high-dose (up to 800 mg/day) CBD on chronic neuropathic pain in SCI.

**Methods:**

This randomised, double-blinded, placebo-controlled, crossover clinical trial was conducted at Neuroscience Research Australia. Adults with SCI and neuropathic pain (≥three months duration) were recruited. Participants were randomised to one of two treatment orders by an unblinded investigator who had no participant contact. Participants and all other investigators were blinded. Participants consumed oral CBD and placebo over two six-week treatment periods separated by a four-week washout. Treatment was titrated up to 800 mg/day of CBD over two-weeks. The primary outcome was change in self-reported pain intensity on a zero (no pain) to ten (worst pain imaginable) Visual Analogue Scale. Statistical comparisons included CBD versus placebo treatment, and pre-treatment (inactive phase) versus on-treatment (active phase). Outcomes were analysed by modified intention-to-treat. The study is registered with anzctr.org.au, ACTRN12622000634774 (not recruiting).

**Findings:**

Forty participants were randomised (August 1, 2022 to December 16, 2024) and 38 included in the primary analysis (n = 6 female). A significant treatment by phase interaction effect (*p* < 0.001) was observed on self-reported pain. Pairwise comparison showed lower pain intensity during the active phase with CBD (mean ± SEM: 3.82 ± 0.23) compared to placebo (mean difference = −0.54, SEM = 0.15, *p* < 0.001), with a 95% confidence interval for the difference of −0.88 to −0.21. Treatments did not differ during the inactive phase (mean difference <0.01, SEM = 0.17, *p* = 1.00, 95% CI = −0.38 to 0.38). Adverse events, nearly all minor, were reported by 68.4% of participants during CBD (*n* = 67 events), and by 52.6% during placebo (*n* = 51 events) treatment.

**Interpretation:**

In this placebo-controlled trial, CBD significantly reduced the self-reported intensity of neuropathic pain and was generally well-tolerated. While modest in magnitude, the observed effect supports further research into high-dose CBD for chronic neuropathic pain.

**Funding:**

Spinal Cord Injury Research Grant NSW Health and the Lambert Initiative for Cannabinoid Therapeutics at The University of Sydney.

**Research in context**

**Evidence before this study**

PubMed, TrialSearch.who.int, ClinicalTrials.gov, ClinicalTrialsRegister.eu, and anzctr.org.au were searched for records between January 1 2020 and November 30 2025, with the following search strings: (“cannabidiol” OR “CBD”) AND (“neuropathic” OR “pain” OR “chronic pain” and combinations) AND (“randomised” and “randomized”). Trials assessing only combinations of cannabidiol (CBD) with other cannabis components and not CBD alone were excluded. To our knowledge, all randomised studies examining the relationship between CBD-only and chronic pain were reviewed. Two randomised trials examined CBD effects on chronic neuropathic pain and reported no significant changes. However, both used doses of CBD far smaller in magnitude than those in the current study.

**Added value of this study**

To our knowledge, this is the first study to examine high-dose CBD efficacy in chronic neuropathic pain. The study also provides data on the adverse events of long-term daily CBD treatment within the context of standard care for neuropathic pain following spinal cord injury (SCI). The study provides preliminary evidence of a beneficial effect of high-dose CBD on neuropathic pain experience, which warrants further investigation in larger clinical trials.

**Implications of all the available evidence**

This trial provides evidence that high-dose CBD is safe and effective in treating chronic neuropathic pain following SCI. Additionally, the study provides initial evidence of a subgroup effect, whereby CBD is more effective in some individuals than others: this also warrants further exploration.

## Introduction

Over two-thirds of people with spinal cord injury (SCI) experience chronic neuropathic pain^1^ which is often so severe that many consider it the most debilitating consequence of their injury.^2^ Current treatments for chronic neuropathic pain are often inadequate: only 40–50% of individuals achieve meaningful (>50%) pain relief, and many therapies have significant side effects.^3^ A substantial number of people with SCI and neuropathic pain report using cannabis and cannabinoids to treat their symptoms, including secondary symptoms such as anxiety and depression.^4^

Cannabidiol (CBD), a non-intoxicating and non-addictive cannabinoid found in *Cannabis sativa* L., has been demonstrated to be a safe and well-tolerated intervention in the treatment of conditions such as paediatric epilepsy, anxiety and psychosis.^5^ In preclinical studies, CBD has also proven effective in reducing neuropathic pain, targeting both the sensory and affective components.^6^^,^^7^ CBD products are currently widely prescribed and used in the community as an intervention for chronic pain despite a lack of compelling supportive evidence of efficacy.^8^

Very few studies to date have examined CBD monotherapy for the treatment of neuropathic pain in humans.^9^, ^10^, ^11^ Two clinical trials using low oral doses of CBD (5–50 mg/day) reported no significant changes in neuropathic pain intensity.^9^^,^^10^ Notably, however, an investigation using a higher oral CBD dose (120 mg/day) reported pain relief superior to placebo.^12^ The clinical effects of CBD in epilepsy, anxiety, psychosis and addiction are most apparent at doses between 300 and 1500 mg^5^, but such dose ranges are yet to be examined in the context of neuropathic pain, representing a significant knowledge gap. This was the focus of the current trial. Importantly, CBD is well tolerated at very high doses (i.e, 1500–6000 mg/day)^13^ so there are a few major safety issues relating to administering higher dose ranges to chronic pain patients.

The primary objective of this study was, therefore, to investigate the effect of a high oral dose of CBD (800 mg/day) on chronic neuropathic pain related to SCI. We hypothesised that CBD would reduce pain intensity compared with a placebo. Additionally, this study measured the effect of CBD on a variety of secondary outcomes, including self-reported quality of neuropathic pain, pain catastrophising, pain impact on quality of life, and self-reported depression, anxiety, stress, and sleep.

## Methods

### Study design

This randomised, double-blind, placebo-controlled, crossover, phase 2 clinical trial was conducted in Sydney, Australia. Ethics approval was obtained from the University of Sydney Human Research Ethics Committee (2021-936), and the trial was prospectively registered with the Australian New Zealand Clinical Trials Registry (ACTRN12622000634774) and completed. No outcomes were modified post-registration. Data analysis began in December 2024, and unblinding occurred May 2, 2025, after publication of the statistical analysis plan online (full script: https://github.com/rebeccavr/SCAN-trial-Statistical-Analysis-Plan).

### Participants

Adults (>18 years) with complete or incomplete SCI and neuropathic pain >3 months were recruited from Prince of Wales Hospital outpatient clinic and SCI advocacy groups via pamphlets. Exclusion criteria included unstable pain medication use, interacting medications, recent cannabis use, substance dependence, positive urine drug screens, major psychiatric illness or suicidality, and pregnancy or lactation. Full criteria are provided in the Supplementary Materials. Sex was measured by self-report, giving options of female, male, unspecified, and rather not say. All participants provided written informed consent.

### Randomisation and masking

Participants were allocated (1:1) to one of two treatment orders using a computer-generated randomisation schedule (10 blocks of four, one block of two), prepared by an unblinded investigator (EC) with no participant contact. All other investigators, site staff, and participants were blinded. Participants were assigned randomisation codes in order of recruitment (i.e, R01, R02, R03), which placed them in either CBD or placebo first arms. The study drug was sent to the dispensing pharmacy (Prince of Wales Hospital pharmacy), blinded (i.e, labelled as per the randomisation schedule). Neither the pharmacist, the researchers, nor the patient knew which order of treatment each bottle contained, as both active treatment and placebo were identical in terms of look, smell, taste, and colour. At the point of analysis, treatments (i.e, CBD or placebo) were allocated a letter (i.e, A or B) by the unblinded researcher (EC). All analysis plans were complete, and the primary outcome was analysed before unblinding occurred.

### Procedures

Eligibility was assessed via an online screening visit by a study physician and researcher. Each participant then attended four in-person sessions: one pre- and one post-treatment for each of two 6-week treatment periods, separated by a 4-week washout. At each visit, urine drug (DrugCheck® NxStep Onsite Urine Drug Test) and pregnancy (where applicable, Human Chorionic Gonadotrophin Cassette, Alere™) testing were performed.

Participants received CBD (200 mg/capsule; purity = 99%) or a matched placebo (BOD Australia Ltd, see Supplementary Materials for details). Orally ingested dosing was 200 mg/day (days 1–4), 400 mg/day (days 5–8), 600 mg/day (days 9–12), and 800 mg/day (days 13–42). Patients were instructed to take the study medication along with a meal to enhance drug bioavailability.^14^ All patients started by taking the medication in the morning, then morning and lunch, and then morning, lunch, and dinner with the highest dose (400 mg) taken at dinner time.

Blood samples were collected to perform genotyping, and plasma samples were analysed in triplicate to measure CBD, tetrahydrocannabinol (THC) and metabolite concentration via a previously validated LCMS/MS method.^15^ Accuracy, precision, linearity, and analytical range are described in Table 1 in Supplementary Materials. Blood was genotyped for two single-nucleotide polymorphisms (SNPs) in the fatty acid amide hydrolase (FAAH) enzyme gene (rs324420 C>A, rs3766246 G>A) under a dominant model. FAAH is a membrane-bound enzyme that degrades key endocannabinoids such as anandamide. The presence of one or more of the SNPs listed above is linked to reduced activity and expression of FAAH, and thereby increased endocannabinoid concentrations, which may impact pain experience and decreased pain perception.^16^^,^^17^

**Table 1:** Baseline participant characteristics.

Characteristic	No. (%)		
	‘CBD–placebo’ (n = 20)	‘placebo–CBD’ (n = 18)	total (n = 38)
Age: mean (SD), range, y	56 (15), 25–78	54 (14), 24–74	55 (14), 24–78
Sex			
Female	2 (10)	4 (22)	6 (16)
Male	18 (90)	14 (78)	32 (84)
Body mass index: mean (SD), range	27, (6), 21–43	28 (6), 17–39	27 (6), 17–43
Time since SCI: mean (SD), range, y	8 (9), 1–34	9 (10), 1–34	9 (9), 1–34
Type of injury^a^			
Tetraplegia	12 (60)	9 (50)	21 (55)
Paraplegia	8 (40)	9 (50)	17 (44)
AIS score			
A	4 (20)	2 (11)	6 (16)
B	2 (10)	4 (22)	6 (16)
C	6 (30)	2 (11)	8 (21)
D	8 (40)	10 (56)	18 (47)
Time since pain onset and randomisation: mean (SD), range, y	8 (9), 1–34	8 (8), 1–24	8 (8), 1–34
Baseline overall pain score: mean (SD), range			
Average of day	4 (2), 0–9	5 (2), 0–8	4 (3), 0–9
Morning	4 (2), 0–9	4 (2), 0–8	4 (2), 0–9
Afternoon	4 (2), 0–9	5 (2), 1–8	5 (2), 0–9
Evening	4 (2), 0–9	5 (2), 1–8	5 (2), 0–9
Baseline pain medications by subclass			
Pain-related medications^b^			
Gabapentinoids	15 (75)	14 (78)	29 (76)
Muscle relaxants	8 (40)	6 (33)	14 (37)
Non-opioid analgesics	5 (25)	9 (50)	14 (37)
Tricyclic antidepressants	5 (25)	8 (44)	13 (34)
Opioids	7 (35)	2 (11)	9 (24)
SSRIs	3 (15)	5 (28)	8 (21)
NSAIDs	3 (15)	4 (22)	7 (18)
Benzodiazepines	1 (5)	4 (22)	5 (13)
Other	1	4	5
Non-pain related medications			
Solifenacin	4 (20)	4 (22)	8 (21)
Docusate sodium	2 (10)	5 (28)	7 (18)
Oxybutynin	3 (15)	2 (11)	5 (13)
Pantoprazole	3 (15)	2 (11)	5 (13)
Apixaban	2 (10)	1 (6)	3 (8)
Ezetimibe	2 (10)	1 (6)	3 (8)
Macrogol	2 (10)	1 (6)	3 (8)
Senna glycoside	1 (5)	2 (11)	3 (8)
Other	65	33	38
Comorbid health conditions			
Cardiovascular	4 (20)	1 (6)	5 (13)
Gastrointestinal	3 (15)	1 (6)	4 (11)
Musculoskeletal (∗ not related to SCI)	0 (0)	3 (17)	3 (8)
Haematopoietic lymphatic	2 (10)	1 (6)	3 (8)
Endocrine-metabolic	1 (5)	0 (0)	1 (3)
Renal genitourinary	1 (5)	0 (0)	1 (3)
Respiratory	1 (5)	0 (0)	1 (3)
Ear, nose, or throat	0 (0)	2 (11)	2 (5)
Malignancy	1 (5)	1 (6)	2 (5)
Genetic disorders	1 (5)	1 (6)	2 (5)
Self-reported past psychiatric history			
Depression disorder	7 (35)	9 (50)	16 (42)
Anxiety disorder	3 (15.0)	4 (22)	7 (18)
Post-traumatic stress disorder	0 (0)	2 (11)	2 (5)
Drug use disorder	0 (0)	1 (6)	1 (3)

AIS: American Spinal Injury Association (ASIA) Impairment Scale; SD: standard deviation; SCI: Spinal Cord Injury. SSRI: serotonin and norepinephrine reuptake inhibitors; n: number; NSAID: non-steroidal anti-inflammatory drugs; y: year(s).

a See eMethods for additional information about inclusion and exclusion criteria. Note: Medication list excludes supplements such as multivitamins, vitamin D, vitamin B, magnesium, calcium, folic acid, iron tablets, potassium chloride, probiotic, melatonin, and salt.

b See Supplementary Table S2 for additional information on sub-class components for pain medication.

### Outcomes

The primary outcome was change in current pain intensity, measured using a Visual Analogue Scale (VAS; 0 = no pain, 10 = worst pain imaginable). Pain ratings were recorded remotely three times daily (08:00, 13:00, 18:00) on alternate weekdays (Monday, Wednesday, and Friday) from 1 week pre-treatment (i.e, the ‘inactive’ phase) through each 6-week treatment (i.e, the ‘active’ phase). Ratings were not collected during washout. Alternate weekdays reporting (rather than daily reporting) was utilised to provide a realistic and manageable participant burden, particularly given the long treatment durations involved in the crossover trial design and the data collection at multiple time points each day.

Secondary outcomes were assessed at each in-person visit (i.e, the visits prior to CBD and placebo treatment commencing and the visits during each treatment): (1) Neuropathic Pain Questionnaire Short Form^18^ (NPQ); (2) Depression Anxiety Stress Scales^19^ (DASS); (3) Pain Catastrophizing Scale^20^ (PCS); (4) State-Trait Anxiety Inventory^21^ (STAI); (5) Brief Pain Inventory (short form)^22^ (BPI); (6) Douleur Neuropathique 4^23^ (DN4); and (7) Pittsburgh Sleep Quality Index short form^24^ (PSQI). Brain imaging and actigraphy outcomes will be published separately. For both primary and secondary outcomes, the inactive phase was defined as the two pre-treatment sessions (i.e, baseline), and the active phase as the time participants consumed CBD and placebo.

Adverse events (AEs) were recorded at the end of each treatment period using a structured checklist and open-ended questioning. The classification of AEs can be found in the Supplementary Materials. Adverse-event rates were calculated as the number of adverse events divided by the number of participants in each treatment arm. Rate ratios were then computed by dividing the CBD rate by the placebo rate, and 95% confidence intervals were derived using standard log-transformed rate-ratio methods.

After each treatment period, participants were asked to guess treatment assignment (CBD, placebo, unsure) and rated their confidence in guessing (Not at all/Somewhat/Moderately/Extremely). The intention to use CBD post-trial was assessed at 4-week follow-up by phone (yes/no/unsure).

### Statistical analysis

A priori power analysis (Cohen's *f* = 0.23) indicated ≥34 participants were required to achieve 90% power (α = 0.05), accounting for 5% unusable data and 5% dropout.^12^

Analyses were conducted in R (v4.4.2) and figures were prepared in CorelDRAW 2021. The primary outcome was analysed with linear mixed-effects models fitted with *lme4* function (*lme4* package). Competing random-effects structures were compared by *AICc* function (MuMIn package), to assess each model's goodness of fit and complexity, followed by selection of fixed effects and interactions (Treatment [categorical: CBD or placebo], Phase [categorical: Active and Inactive], Day [continuous], and their combinations). Additional covariates (Time of Day [categorical: Morning, Afternoon and Evening], Treatment Order [Categorical: CBD First and Placebo First], Treatment Arm [categorical: Arm 1 and Arm 2]) were included for all study outcomes and only retained if they improved model fit (i.e, reduced the value of the Akaike's Information Criterion corrected for small sample sizes). Model assumptions were checked with the *check_model* function (performance package), assessing linearity, normality of residuals, homogeneity of variance, and multicollinearity. Main/interaction effects were assessed with type III ANOVA using the *anova* function and significant effects were followed by Dunn-Šidák corrected post hoc tests using the *emmeans* function (emmeans package).

Secondary outcomes followed the same approach, except models always included Treatment, Phase, and Treatment × Phase. Positively skewed outcomes were square-root or log-transformed if diagnostics indicated violation of assumptions and the dependent variable comprised only positive (non-zero) values.

A post-hoc responder analysis was conducted to further examine individual pain responses. Responders and non-responders were classified by comparing self-reported pain during the final week of CBD active treatment with the baseline observed during the corresponding inactive phase. Cumulative distribution functions of percent pain reduction were plotted for each treatment, and responder proportions were calculated at the IMMPACT thresholds of ≥10%, ≥20%, ≥30%, and ≥50% improvement in pain during the final week of CBD treatment compared to baseline. Differences in responder rates between responder thresholds (≥10%, ≥20%, ≥30%, and ≥50%) were compared using χ^2^ or Fisher's exact tests, as appropriate. Additionally, to explore whether responders (≥30%) showed greater improvements in secondary outcomes, pre- and post-treatment values for CBD were used to derive change scores for each measure. Normality of residuals was assessed, and either independent-samples t-tests or Wilcoxon rank-sum tests were applied as appropriate. Pain reduction during CBD treatment was correlated with clinical measures (i.e, American Spinal Injury Association Impairment Scale (ASI) score, time since injury, and time since pain onset, type of injury) using Spearman rank correlation and Kruskal–Wallis tests. Pearson correlation tests and simple linear regression models were used to examine the relationship between pain reduction during CBD treatment and plasma concentrations of CBD and its metabolites (6-OH-CBD, 7-OH-CBD, 7-COOH-CBD).

Genotype–response associations (≥30% pain reduction) were tested using logistic regression under a dominant model (A allele vs non-carriers). Odds ratios with 95% CIs were estimated separately for CBD and placebo arms.

Statistical significance was set at p < 0.05. Reported values are estimated marginal means ± standard error. Visualisation was performed using the function *ggplot()* from the package “ggplot2”.

### Role of the funding source

The funder of the study had no role in study design, data collection, data analysis, data interpretation, or writing of the report.

## Results

**Fig. 1:**
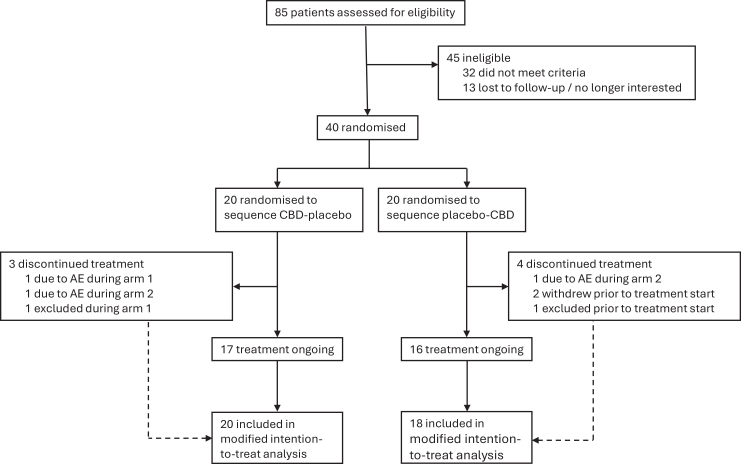
**Trial profile.** Flow diagram of participation in the trial. Crossover was performed in both groups, as illustrated by the CBD-placebo and placebo-CBD labels. CBD: Cannabidiol; AE: adverse event.

Of the 40 participants who consented between August 1, 2022–December 16, 2024, one was excluded before the first visit (urine drug test positive for THC), and one was lost to follow-up, leaving 38 included in the modified intention-to-treat primary outcome analysis (95.0%) (Fig. 1). Thirty-seven participants (92.5%) completed secondary outcome measures (1 withdrew from questionnaires).

Compliance with daily pain ratings completion was high: the mean completion rate was 98%, with 35 of 37 participants (94.6%) submitting data on ≥80% of days. Compliance with secondary outcome reporting averaged 85%, with 30 of 37 (81.1%) submitting ≥70% of assessments.

Baseline characteristics are shown in Table 1. Of the 38 participants included, 32 (84.2%) were male, with a mean (SD) age of 54.9 (14.3) years; 21 (55.3%) had tetraplegia, and 18 (47.4%) had an AIS D classification. Five participants (13.2%) reported cardiovascular disease, and 16 (42.1%) reported a history of depression. The mean (SD) number of pain medications was 2.4 (1.7) (range, 0–7). Common analgesics/antispasmodics included gabapentinoids (*n* = 29, 76.3%), paracetamol (*n* = 14, 36.8%), baclofen (*n* = 14, 36.8%), tricyclic antidepressants (*n* = 13, 34.2%), and opioids (*n* = 11, 29.0%) (Supplementary Materials Table 2). Common non-analgesic medications included solifenacin (*n* = 8, 21.1%), docusate sodium (*n* = 6, 15.8%), oxybutynin (*n* = 5, 13.2%), and pantoprazole (*n* = 5, 13.2%).

**Fig. 2:**
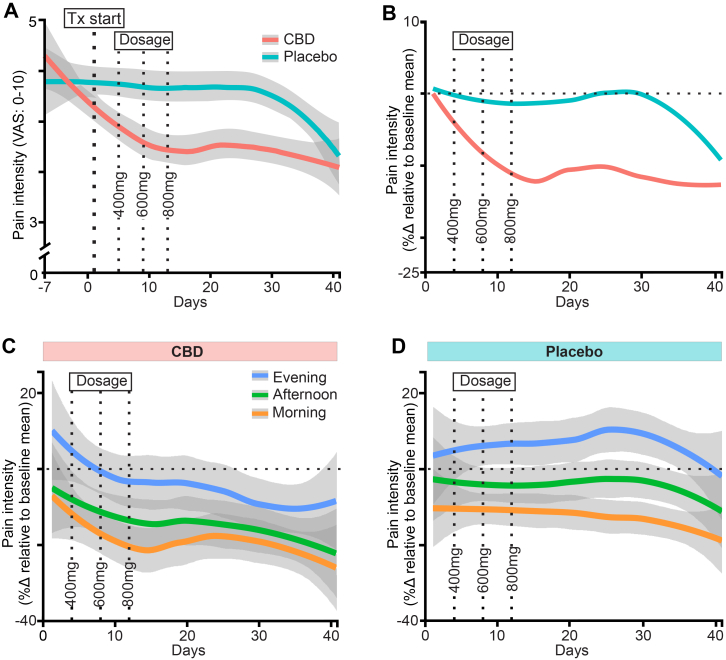
**Daily pain intensity during the cannabidiol (CBD) and placebo treatment periods.** Orally ingested dosing was 200 mg/day (days 1–4), 400 mg/day (days 5–8), 600 mg/day (days 9–12), and 800 mg/day (days 13–42). **(A)** Absolute pain intensity on a 11-point Visual Analogue Scale (VAS), **(B)** Percent change in pain intensity relative to the 1-week inactive baseline period for each treatment, **(C**–**D)** Percent change in pain intensity relative to the pre-treatment (“inactive”) baseline period, stratified by time of day for the **(C)** CBD and **(D)** placebo treatments. In all panels, solid lines represent the mean and shaded areas represent the standard error. Vertical dotted lines indicate dose escalations. Tx indicates treatment.

Overall pain scores were significantly influenced by the interaction between Treatment and Phase (*F*(1, 3887.12) = 28.58, *p* < 0.001; Table 2 and Supplementary Materials Table S3). Specifically, participants reported lower pain intensity during the active Phase of CBD treatment (mean ± SEM: 3.82 ± 0.23; Table 2) compared to the inactive Phase (4.53 ± 0.23, *p* < 0.001), and compared to the active (4.36 ± 0.25, mean difference = −0.54, SEM = 0.15, 95% CI = −0.88 to −0.21, *p* < 0.001) and inactive (4.53 ± 0.26, *p* < 0.001; Table 2) Phases of placebo treatment (Fig. 2). No difference between treatments was observed during the inactive Phase (mean difference <0.01, SEM = 0.17, 95% CI = −0.38 to 0.38, *p* = 1.00; Table 2).

**Table 2:** Model effects and estimated marginal means for the overall pain primary and secondary outcomes defined by their significant effect measured using linear mixed effect models.

Outcome	Effect	p-value	Treatment	Phase	LSMean	SE	95% CI
Overall pain	Treatment	0.083	CBD	Active	3.82	0.23	3.38–4.26
	Phase	**<0.001∗∗∗**	Placebo	Active	4.36	0.25	3.87–4.85
	Time	**<0.001∗∗∗**	CBD	Inactive	4.53	0.23	4.07–4.99
	Treatment x Phase	**<0.001∗∗∗**	Placebo	Inactive	4.53	0.26	4.02–5.03
NPQ	Treatment	0.55	CBD	Active	−0.15	0.14	−0.43–0.13
	Phase	0.17	Placebo	Active	0.072	0.15	−0.22–0.37
	Treatment x Phase	**0.0048∗∗**	CBD	Inactive	0.26	0.14	−0.013–0.53
			Placebo	Inactive	−0.08	0.15	−0.37–0.21
DASS Depression	Treatment	**0.044∗**	CBD	Active & Inactive	9.26	1.45	6.42–12.10
	Phase	0.88	Placebo	Active & Inactive	7.10	0.96	5.22–8.98
	Treatment x Phase	0.81					
DASS Anxiety	Treatment	0.56	CBD	Active & Inactive	3.55	0.60	2.37–4.73
	Phase	**0.022∗**	Placebo	Active & Inactive	4.41	0.60	3.23–5.59
	Treatment x Phase	0.82					

EMM, Model-Estimated Mean; SE, Standard Error; Min, Minimum; Max, Maximum. Note, Significant *p*-values appear in bold. ∗p < 0.05. ∗∗p < 0.01. ∗∗∗p < 0.001.

A main effect of Phase was observed (*F*(1, 3886.68) = 74.10, p < 0.001) across the treatments, with lower pain during active Phases (4.09 ± 0.23) than inactive Phases (4.53 ± 0.23; mean difference = −0.44, SEM = 0.051, p < 0.001). Pain also varied by Time of Day (*F*(2, 3878.98) = 142.69, *p* < 0.001), increasing from morning (3.95 ± 0.23) to afternoon (4.29 ± 0.23), and peaking in the evening (4.69 ± 0.23). Morning pain was significantly lower than both afternoon (−0.34, SEM = 0.043, *p* < 0.001) and evening pain (−0.74, SEM = 0.044, *p* < 0.001), and afternoon pain was lower than evening pain (−0.40, SEM = 0.044, *p* < 0.001). There was no significant main effect of Treatment (*F*(1, 37.29) = 3.17, *p* = 0.083) across the phases.

When comparing percent change in pain between the baseline week and the final week of treatment, an average of 14.0% reduction in pain in the last week was seen with CBD and a 6.5% reduction with placebo. Responder analyses were employed, which initially involved the identification of participants showing clinically meaningful improvement with CBD compared with placebo treatment. A significantly greater proportion of participants receiving CBD achieved a ≥30% reduction in pain (37.8% vs 11.1%; *p* = 0.014), whereas responder rates at the ≥10%, ≥20%, and ≥50% thresholds did not differ significantly between responder and non-responder groups (Supplementary Material Fig. S1 and Table S4).

A significant positive association was found between pain reduction during CBD treatment and time since injury (ρ = 0.38, *p* = 0.021, 95% CI: 0.06 to 0.63) and time since pain onset (ρ = 0.39, *p* = 0.017, 95% CI: 0.08 to 0.64; Supplementary Material Table S5), with individuals having longer-standing injury/pain tending to experience a greater pain reductions. No association was observed between pain reduction with CBD and AIS impairment scores and injury type (i.e, paraplegia or tetraplegia; Supplementary Material Table S5). Pearson correlations, scatterplots with LOWESS smoothing and linear regression overlays showed no significant association between plasma CBD concentrations (or their metabolites) and pain reduction (Supplementary Material Table S6).

Responders showed a greater reduction in BPI intensity compared with non-responders (mean difference −1.12, 95% CI: −1.86 to −0.38; *p* = 0.008; Supplementary Materials Table S7), indicating a modest improvement in pain severity among those who responded to CBD. No association was seen in the DASS, DN4, PCS, BPI interference, STAI, NPQ, PSQI, PSC scores, and rate of incidence of co-morbid health conditions (e.g, cardiovascular, gastrointestinal, respiratory) when comparing ≥30% responder and non-responder (Supplementary Materials Table S8).

NPQ scores were significantly affected by the interaction between Treatment and Phase (*F*(1, 78.97) = 8.41, *p* = 0.0048; Table 2 and Supplementary Materials Table S3, Fig. S3). Post hoc comparisons showed that participants reported significantly higher NPQ scores during the inactive Phase of CBD (0.27 ± 0.14) compared to the inactive placebo Phase (−0.08 ± 0.15, mean difference = −0.35, SEM = 0.14, 95% CI = −0.67 to −0.019, *p* = 0.036). No significant difference was seen when comparing the active Phase of CBD (−0.15 ± 0.14) to the active phase of placebo (0.07 ± 0.15, mean difference = 0.23, SEM = 0.14, 95% CI = −0.092 to 0.54, *p* = 0.21; Supplementary Materials Table S3).

**Fig. 3:**
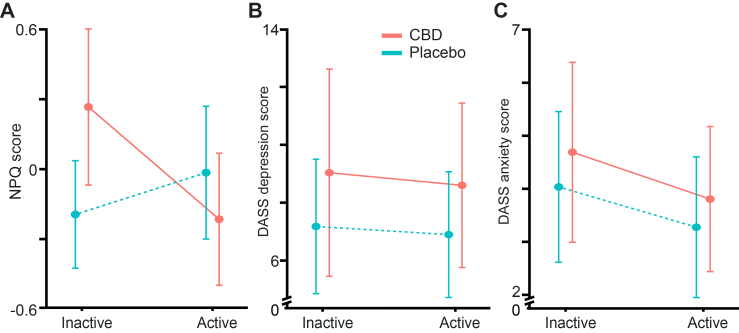
**Changes in neuropathic pain, depression, and anxiety scores from baseline to end of treatment.** Mean scores with 95% CIs are shown for **(A)** Neuropathic Pain Questionnaire (NPQ), **(B)** Depression Anxiety Stress Scale (DASS)—Depression Subscale, **(C)** DASS—Anxiety Subscale. Scores were measured at baseline (inactive phase) and at the end of the 6-week treatment period (active phase). Dotted lined represent the placebo group.

DASS-Anxiety scores revealed a significant main effect of Phase (*F*(1, 57.15) = 5.52, *p* = 0.022; Table 2 and Supplementary Materials Table S3), with lower DASS-Anxiety scores reported during active Phases (3.55 ± 0.60) overall than inactive Phases (4.41 ± 0.60, mean difference = −0.86, SEM = 0.37, *p* < 0.023; Fig. 3). In contrast, DASS-depression scores showed a main effect of Treatment (*F*(1, 32.79) = 4.38, *p* = 0.044) with higher scores observed on CBD overall (9.26 ± 1.45; Supplementary Materials Table S3) compared to placebo (7.10 ± 0.96, mean difference = 2.16, SEM = 1.04, *p* = 0.046) (i.e, irrespective of Phase). No significant main effects or interactions were found for DASS stress, DN4, PCS, BPI, STAI PSQI or PSC Rumination scores (all *p*-values>0.05; Supplementary Materials Table S3).

A total of 119 AEs were reported (Supplementary Materials Table S9). Sixty-seven AEs (56.3%) were reported by 26 participants during CBD treatment, 51 (42.9%) by 20 participants during placebo, and 1 (0.8%) during washout from CBD. The AE rate was 2.58 events per participant during CBD and 2.55 during placebo, yielding a rate ratio of 1.01 (95% CI: 0.70 to 1.45). Two participants withdrew due to mild AEs: one for nausea (CBD arm) and one for multiple symptoms (placebo arm), including spasticity, stiffness, diarrhoea, and fatigue (Fig. 1).

The most frequent AEs during CBD were unusual tiredness/sleepiness (*n* = 15), nausea (*n* = 10), diarrhoea (*n* = 7), feeling unwell (*n* = 7), loss of appetite (*n* = 6), and stomach pain/discomfort (*n* = 5). During placebo, the most frequent were unusual tiredness/sleepiness (*n* = 15), feeling unwell (*n* = 9), nausea (*n* = 6), and diarrhoea (*n* = 5). AEs deemed likely related to CBD included unusual tiredness/sleepiness (*n* = 11), nausea (*n* = 6), diarrhoea (*n* = 3), loss of appetite (*n* = 3), stomach pain/discomfort (*n* = 3), itchiness (*n* = 2), insomnia (*n* = 1), and indigestion (*n* = 1), with similar events also observed during placebo (Supplementary Materials Table S10). The mean (SEM) time to onset of these likely linked AEs was earlier with CBD (14.0 [4.4] days) than with placebo (26.7 [9.6] days).

Three non-mild AEs occurred: 2 during CBD (i.e, urinary tract infection resolving with IV antibiotics and femur fracture from a home transfer, the latter leading to dropout) and 1 during post-CBD washout (i.e, stroke following hip dislocation while surfing, triggering autonomic dysreflexia; resumed the trial after making a full recovery). None were deemed related to treatment, and all participants recovered.

In the 34 participants who provided blood samples, CBD and its metabolites (6-OH-CBD, 7-OH-CBD, 7-COOH-CBD) were present only after CBD treatment, with residual levels observed before arm 2 in participants who received CBD first (Supplementary Materials Figs. S2 and S3). No THC or THC metabolites were present in samples at any timepoint.

A total of 35 participants completed the expectations questionnaire (CBD, *n* = 34; placebo, *n* = 32). During the CBD treatment phase, 18 (52.9%) guessed correctly, 16 (47.1%) guessed incorrectly, and 1 (2.9%) was unsure. For the placebo, 25 (78.1%) guessed correctly, 4 (12.5%) incorrectly, and 4 (12.5%) were unsure. The placebo guesses were generally more accurate than CBD guesses. Accuracy also varied by confidence level (Supplementary Materials Table S11).

Among the 34 participants completing follow-up, just over half reported intending to seek a CBD prescription. This group generally guessed their treatment more accurately than those who did not, although placebo guesses remained most accurate overall (Supplementary Materials Table S12).

Of the 33 participants who completed genotyping, no significant association (*p* > 0.05) was found between FAAH polymorphisms and treatment response (Supplementary Materials Table S13).

## Discussion

This randomised, double-blinded, placebo-controlled, crossover trial (RCT) provides, for the first time, evidence that CBD, taken at a relatively high dose (800 mg/day), can reduce self-reported pain intensity in individuals with chronic neuropathic pain. The effect was statistically different from the reduction in pain intensity reported with the placebo treatment. Secondary outcomes, including other pain and psychological measures, were largely unchanged by CBD. Notably, CBD treatment was well-tolerated, with most AEs minor and similar to those observed during placebo. This is consistent with the benign side effect profile of CBD reported across many clinical trials involving adult participants.^13^

Our results align with those of preclinical studies^6^^,^^7^ and two open-label clinical trials^12^^,^^25^ suggesting the efficacy of CBD in neuropathic pain. They diverge, however, from two recent RCTs that found no significant amelioration of chronic neuropathic pain with CBD, albeit at much lower doses (<50 mg/day).^9^^,^^10^ These overall outcomes are consistent with recent conclusions that CBD has few, if any, significant therapeutic effects at doses <300 mg/day.^26^ Visual inspection of our data (Fig. 2) suggests that pain intensity decreased as the CBD dose was titrated upwards over the treatment period, up to the highest dose (800 mg/day). Preclinical studies have likewise shown that doses of 5–20 mg/kg CBD (administered orally, intravenously and intraperitoneally) are required to relieve pain-related allodynia and hyperalgesia in rats.^6^^,^^7^ The corresponding human equivalent oral dose is ∼340–1362 mg, which is within the dose range of the current study.^27^ Notably, we observed a significant effect of time of day with respect to pain intensity, highlighting that multiple pain measures throughout the day and over the course of a clinical trial are desirable in providing robust efficacy assessment.

While CBD appeared effective in reducing pain intensity, the average reduction (14%) was below that considered to be moderately clinically significant (30%)^28^ and more consistent with a minimal clinically important change. Nonetheless, 14/37 participants (37.8%) achieved pain intensity reductions at or above the moderate clinically significant level, highlighting that CBD can be clinically effective in a proportion of individuals with chronic neuropathic pain. Importantly, among participants who did not demonstrate clinically significant improvements, 10 indicated they would seek an ongoing CBD prescription post-trial.

Anxiety, depression and poor sleep commonly co-occur with chronic neuropathic pain in SCI^29^^,^^30^ and some emerging evidence indicates that CBD can be an effective intervention for these conditions.^31^ However, in the current study, we found no effect of CBD on measures of depression or quality of life, and pain interference and self-reported anxiety were reduced following treatment with both CBD and placebo. Additionally, we found no difference between responders and non-responders to CBD in secondary outcomes such as depression, anxiety, stress, and quality of life. Importantly, however, mean baseline depression and anxiety scores on the DASS were in the subclinical range, which would make any anxiolytic or antidepressant effect of CBD difficult to identify. In terms of clinical profiles, we observed a link between longer-standing injuries and chronicity of pain and the amount of pain reduction with CBD. It may be that quality of life and mental health factors do not influence the efficacy of CBD on an individual level to the same extent as chronicity of injury and pain.

Several limitations are noted. First, participants were taking a wide range of concomitant medications, which were not deemed ethical to discontinue. As CBD may interact with commonly prescribed analgesics, potential drug–drug interactions may have influenced pain outcomes.^32^ Second, residual CBD and its metabolites were present before the second treatment arm in participants who received CBD first, potentially confounding results. However, statistical methods accounted for order effects, and post-washout CBD plasma concentrations were <15 ng mL^−1^ and unlikely to have any therapeutically relevant effects, given that doses <300 mg appear to lack efficacy in any condition.^26^ Although 7-COOH-CBD concentrations were also elevated, it is unlikely that this metabolite has any physiological activity, given that the analogous THC terminal metabolite is inactive.^33^ Thirdly, participant accuracy of guesses of whether they were taking the CBD treatment was around 50% or chance. However, the possibility that participants had lower expectations of pain change during the placebo treatment cannot be ruled out: 78% of participants correctly guessed that they were taking a placebo, around 30% more accurate than in other CBD and pain studies.^9^^,^^10^ Finally, while this study attempted to offer insights into the mechanisms of action of CBD, our genotyping analysis did not provide many insights due to the small sample size. Therefore, larger trials and mechanism-focused studies are required to better understand how CBD produces pain relief.

In conclusion, CBD, taken at the relatively high oral dose of 800 mg/day, significantly reduced the intensity of chronic neuropathic pain related to SCI. Further RCTs of high-dose CBD in chronic neuropathic pain conditions appear warranted.

## Contributors

Conceptualisation: AS, EC, IM, KK, LAH, NM, RR, SS.

Investigation, Methodology: AP, AS, CBR, CM, DM, FTM, IM, KK, LAH, LS, RG, RR, SS.

Writing—original draft: LAH, RR.

Writing—review & editing: All authors.

Data curation, Formal analysis, Software, Validation & Visualisation: LAH, IM, AP, DM, LS, RG, RR.

Accessed and verified the data: LAH, IM, AP, DM, LS, RG, RR.

Funding acquisition: EC, IM, KK, LAH, SS.

Project administration & Resources: AP, AS, CBR, CM, DM, FTM, IM, KK, LAH, LS, RR, RG, SS.

Supervision: IM, LAH.

All authors have read and approved the final version of the manuscript.

## Data sharing statement

Data are available and consist of deidentified participant information. They can be accessed upon request by emailing luke.henderson@sydney.edu.au and will be available at the time of publication. Access is limited to researchers who provide a methodologically sound proposal, and there are no restrictions on the type of analysis that may be conducted. A signed data access agreement is required for use.

## Declaration of interests

IM received support from the Lambert Initiative for Cannabinoid Therapeutics and NSW Health to conduct this study and holds other research grants from NHMRC/MRFF. IM has been paid for educational webinars run by Althea Limited and has provided expert testimony in multiple medicolegal cases related to cannabis and cannabinoids. IM has several patents to Kinoxis Therapeutics issued and pending, including US12458648, US11890287, US11033555B2, US11491165B2, WO2019227167, WO2019071302, US18547377, and US20220288060A1. IM also holds stock options in Kinoxis Therapeutics. EC received support from an NSW Health grant and from the Lambert Initiative for Cannabinoid Therapeutics for this study. EC contributed to this work while employed at the University of Sydney and is now employed by Aspeya, while maintaining an independent affiliate appointment at the University of Sydney under which contributions to this manuscript were made. Aspeya had no involvement in the design, analysis, interpretation, or writing of this work. All other authors report no competing interests.
