# Supplementary material for: High-dose cannabidiol for chronic neuropathic pain associated with spinal cord injury: a randomised clinical trial
Source: eClinicalMedicine. 2026 May 28;96:103986. doi: 10.1016/j.eclinm.2026.103986 (PMC13233594; doi:10.1016/j.eclinm.2026.103986)
Supplement: Supplementary Materials [file mmc1.docx]

**Supplementary Materials**

Robertson at al; High-dose cannabidiol for chronic neuropathic pain: A randomised clinical trial associated with spinal cord injury

Contents

[1) Inclusion criteria 3](#_Toc228543081)

[2) Exclusion criteria 3](#_Toc228543082)

[3) Removed exclusion criteria 4](#_Toc228543083)

[4) Investigational product 4](#_Toc228543084)

[5) Classification of Adverse Events 4](#_Toc228543085)

[6) Table 1 6](#_Toc228543086)

[7) Table 2 7](#_Toc228543087)

[8) Table 3 8](#_Toc228543088)

[9) Table 4 11](#_Toc228543089)

[10) Figure 1 12](#_Toc228543090)

[11) Table 5 13](#_Toc228543091)

[12) Table 6 14](#_Toc228543092)

[13) Table 7 15](#_Toc228543093)

[14) Table 8 16](#_Toc228543094)

[15) Table 9 17](#_Toc228543095)

[16) Table 10 18](#_Toc228543096)

[17) Figure 2 19](#_Toc228543097)

[18) Figure 3 20](#_Toc228543098)

[19) Table 11 21](#_Toc228543099)

[20) Table 12 22](#_Toc228543100)

[21) Table 13 23](#_Toc228543101)

[22) Research Protocol and Statistical Plan 24](#_Toc228543102)

### **Inclusion criteria**

SCI participants complete or incomplete determined using the ASIA scale;

Below-level neuropathic pain minimum 3 months duration;

Adult and provide informed consent; and

- 1. Proficient in English (must not require an English translator).

### **Exclusion criteria**

- 1. If on pain relieving medications must be on stable dose for at least 6 weeks prior to beginning our trial. This includes all prescription medications for pain including opioids or adjuvant pain medications such as Pregabalin, Amitriptyline and SSRIs. Paracetamol and over-the-counter NSAIDs (e.g., ibuprofen, aspirin, naproxen) within the recommended adult dosage permitted as needed. If there has been recent dose titration then we would wait for a stable 6-week period before entering the individual into the trial.
  2. If on pain relieving medications, they must not be changed in dosage during the trial period;
  3. Cannabis or any other drug or alcohol dependence, as per the International Statistical Classification of Diseases 10th Revision (ICD)-10 criteria or at a medical doctor’s discretion;
  4. Reported use of cannabis within the past 3 months (or at the medical officer’s discretion);
  5. Urine drug test positive for drugs (cannabis – THC only, meth/amphetamines, and cocaine) at each visit;
  6. A history of (self-reported) allergic reaction (e.g. rhinitis, urticaria, contact dermatitis, anaphylaxis) to cannabis, cannabis products or cannabinoids;
  7. A history of (self-reported) a clinically significant adverse response to CBD;
  8. Use of medication(s) that may influence CBD metabolism (e.g. inducers or inhibitors of the CYP450 enzyme system) as determined by the medical doctor;
  9. A history of a major psychiatric disorder within the previous 12 months except for clinically managed anxiety and/or depression as determined via clinical interview using the Diagnostic and Statistical Manual of Mental Disorders (DSM)-V criteria or at the medical doctor’s discretion;
  10. History of suicide attempt or current suicide ideation as determined via clinical interview with the medical doctor;
  11. Pregnancy or lactation - women shall be advised to use reliable contraception for the duration of drug therapy and a urine pregnancy test will be performed where necessary.

### **Removed exclusion criteria**

1. Unable to undergo MRI brain imaging as identified via 'NeuRA MRI Safety Screening Questionnaire' (Appendix 16);
2. Patients with quadriplegia secondary to cervical spine injury.

### **Investigational product**

The investigational product, Bod Australia Limited Bod ECS100, was a pure CBD product manufactured and finished by Gelpell AG (Kirchbergerstrasse 10 CH-9534 Gähwil, Switzerland), the 99% Pure Crystalline CBD Isolate solution was supplied in bulk for encapsulating as finished goods from Linnea SA (Via Cantonale, CH-6595 Riazzino (TI), Switzerland). The manufacturer had no clinical involvement in the project.

### **Classification of Adverse Events**

The assignment of the causality was made by the investigators using the definitions below:

***Unrelated:*** Sufficient information exists to indicate that the aetiology is unrelated to the study drug.

***Unlikely:*** When there is no reasonable temporal association between the study drug and the suspected AE. The event could have been related to the patient’s clinical state or concomitant treatment(s).

***Possible:*** There is some evidence to suggest a causal relationship (e.g., because the event occurs within a reasonable time after the administration of the trial medication). However, the influence of other factors may have contributed to the events (e.g., the participant’s clinical condition, other concomitant treatments).

***Definitely:*** There is clear evidence to suggest a causal relationship, based on:

- Temporal relationship to the administration of CBD;
- Known or response pattern of the suspected CBD;
- Improvement of the event after de-challenge or dosage reduction of the CBD;

The event reappears after repeated exposure (re-challenge).

***Not Assessable:*** When causality is, for one reason or another, not accessible, e.g., because of insufficient evidence, conflicting data or poor documentation.

### **Table 1** Analytical range, Quality Control material and assessment of Standard Deviation, Precision (CV), Accuracy, and Linearity, aggregate across two sets per five batches of sampling (n=10).

| **Analyte** | **Analytical range (ng/mL)** | **QC level** | **QC (ng/mL)** | **Mean conc ± SD** | **CV(%)** | **Accuracy range %** | **Linearity (r²)** |
| --- | --- | --- | --- | --- | --- | --- | --- |
| **6-OH-CBD** | 1.25-150 | QCL | 3 | 3.1 ± 0.13 | 4.1 | 94.3-108.7 | 0.9992 |
|  |  | QCM | 75 | 76.2 ± 3.54 | 4.6 | 93.7-106.3 |  |
|  |  | QCH | 135 | 140.0 ± 5.43 | 3.9 | 94.4-111.0 |  |
| **7-OH-CBD** | 1.25-250 | QCL | 7.5 | 7.6 ± 0.27 | 3.6 | 97.1-105.9 | 0.9994 |
|  |  | QCM | 125 | 116.9 ± 3.28 | 2.8 | 90-96.4 |  |
|  |  | QCH | 225 | 232.9 ± 17.79 | 7.6 | 89.9-109.6 |  |
| **7-COOH-CBD** | 12.5-2500 | QCL | 75 | 78.0 ± 4.10 | 4.1 | 98.2-111.1 | 0.9994 |
|  |  | QCM | 1250 | 1212.6 ± 8.45 | 8.4 | 96.7-104.4 |  |
|  |  | QCH | 2250 | 2189.1 ± 187.41 | 8.6 | 87.8-105.9 |  |
| **CBD** | 1.25-250 | QCL | 7.5 | 7.6 ±0.54 | 7.1 | 91.0-113.4 | 0.9986 |
|  |  | QCM | 125 | 128.6 ± 7.06 | 6.1 | 94.1-109.2 |  |
|  |  | QCH | 225 | 231.7 ± 14.46 | 6.2 | 93.8-112.8 |  |

### **Table 2** Frequency of use of concomitant pain medications.

| **Subclassification** | **Pain medication** | **Total (n)** |
| --- | --- | --- |
| Gabapentinoids | Pregabalin | 20 |
|  | Gabapentin | 9 |
| Opioids | Tapentadol | 5 |
|  | Codeine | 2 |
|  | Oxycodone | 2 |
|  | Buprenorphine | 2 |
| Non-opioid analgesic | Paracetamol | 14 |
| TCAs | Amitriptyline | 8 |
|  | Nortriptyline | 5 |
| Muscle relaxant | Baclofen | 14 |
| SNRIs | Duloxetine | 5 |
|  | Venlafaxine | 2 |
|  | Desvenlafaxine | 1 |
| Benzodiazepines | Diazepam | 5 |
| NSAIDs | Celecoxib | 3 |
|  | Diclofenac | 3 |
|  | Ibuprofen | 1 |
|  | Meloxicam | 1 |
| Gout management | Allopurinol | 2 |
| Capsaicin | Capsaicin | 1 |
| Nutraceutical | Palmitoylethanolamide | 1 |
| Dopamine agonist | Pramipexole | 1 |
| Antipsychotics | Quetiapine | 1 |
| Melatonergic antidepressants | Agomelatine | 1 |

### **Table 3** Model effects and estimated marginal means for secondary outcomes measured using linear mixed effect models.

| **Outcome** | **Effect** | **p‑value** | **Treatment** | **Phase** | **LSMean** | **95% CI** |
| --- | --- | --- | --- | --- | --- | --- |
| **PCS Rumination** | Treatment | 0·052 | CBD | Active | 5·25 | 3·87–6·63 |
|  | Phase | 0·21 | Placebo | Active | 4·25 | 2·86–5·64 |
|  | Order | 0·52 | CBD | Inactive | 5·68 | 4·29–7·08 |
|  | Treatment x Phase | 0·80 | Placebo | Inactive | 4·90 | 3·50–6·30 |
|  | Treatment x Order | 0·065 | — | — | — | — |
|  | Phase x Order | 0·71 | — | — | — | — |
|  | Treatment x Phase x Order | 0·24 | — | — | — | — |
| **PCS Magnification** | Treatment | 0·88 | CBD | Active | 2·42 | 1·50–3·34 |
|  | Phase | 0·28 | Placebo | Active | 2·35 | 1·42–3·27 |
|  | Treatment x Phase | 0·90 | CBD | Inactive | 2·66 | 1·74–3·58 |
|  |  |  | Placebo | Inactive | 2·65 | 1·72–3·57 |
| **PCS Helplessness** | Treatment | 0·13 | CBD | Active | 7·36 | 5·56–9·15 |
|  | Phase | 0·18 | Placebo | Active | 6·33 | 4·53–8·13 |
|  | Order | 0·39 | CBD | Inactive | 7·83 | 6·02–9·64 |
|  | Treatment x Phase | 0·68 | Placebo | Inactive | 7·24 | 5·42–9·05 |
|  | Treatment x Order | 0·98 | — | — | — | — |
|  | Phase x Order | 0·74 | — | — | — | — |
|  | Treatment x Phase x Order | 0·66 | — | — | — | — |
| **PCS Total Score** | Treatment | 0·076 | CBD | Active | 15·1 | 11·39–18·80 |
|  | Phase | 0·11 | Placebo | Active | 13·0 | 9·29–16·70 |
|  | Order | 0·62 | CBD | Inactive | 16·2 | 12·48–19·90 |
|  | Treatment x Phase | 0·69 | Placebo | Inactive | 14·9 | 11·13–18·60 |
|  | Treatment x Order | 0·44 | — | — | — | — |
|  | Phase x Order | 0·73 | — | — | — | — |
|  | Treatment x Phase x Order | 0·92 | — | — | — | — |
| **STAI Total (SQRT)** | Treatment | 0·75 | CBD | Active | 7·83 | 7·35–8·31 |
|  | Phase | 0·91 | Placebo | Active | 7·71 | 7·22–8·19 |
|  | Treatment x Phase | 0·50 | CBD | Inactive | 7·74 | 7·25–8·22 |
|  |  |  | Placebo | Inactive | 7·78 | 7·29–8·26 |
| **STAI State (SQRT)** | Treatment | 0·44 | CBD | Active | 5·25 | 4·92–5·58 |
|  | Phase | 0·87 | Placebo | Active | 5·09 | 4·75–5·42 |
|  | Treatment x Phase | 0·45 | CBD | Inactive | 5·19 | 4·85–5·52 |
|  |  |  | Placebo | Inactive | 5·18 | 4·85–5·51 |
| **STAI Trait (SQRT)** | Treatment | 0·88 | CBD | Active | 5·90 | 5·62–6·18 |
|  | Phase | 0·69 | Placebo | Active | 5·88 | 5·60–6·16 |
|  | Treatment x Phase | 0·65 | CBD | Inactive | 5·84 | 5·56–6·12 |
|  |  |  | Placebo | Inactive | 5·89 | 5·60–6·17 |
| **BPI Intensity** | Treatment | 0·74 | CBD | Active | 4·26 | 3·74–4·78 |
|  | Phase | 0·48 | Placebo | Active | 4·13 | 3·60–4·65 |
|  | Treatment x Phase | 0·19 | CBD | Inactive | 3·99 | 3·47–4·51 |
|  |  |  | Placebo | Inactive | 4·21 | 3·68–4·73 |
| **BPI Interference** | Treatment | 0·70 | CBD | Active | 4·73 | 3·76–5·69 |
|  | Phase | 0·18 | Placebo | Active | 4·66 | 3·72–5·59 |
|  | Treatment x Phase | 0·54 | CBD | Inactive | 4·06 | 3·13–4·99 |
|  |  |  | Placebo | Inactive | 4·40 | 3·44–5·37 |
| **BPI Interference (Non‑Walking)** | Treatment | 0·61 | CBD | Active | 4·77 | 4·00–5·54 |
|  | Phase | 0·47 | Placebo | Active | 4·55 | 3·78–5·32 |
|  | Treatment x Phase | 0·15 | CBD | Inactive | 4·24 | 3·47–5·02 |
|  |  |  | Placebo | Inactive | 4·73 | 3·94–5·51 |
| **DASS Stress** | Treatment | 0·21 | CBD | Active | 8.45 | 5.86–11.0 |
|  | Phase | 0·30 | Placebo | Active | 7.54 | 4.92–10.2 |
|  | Treatment x Phase | 0·93 | CBD | Inactive | 9.06 | 6.44–11.7 |
|  |  |  | Placebo | Inactive | 8.27 | 5.63–10.9 |
| **PSQI Global Score** | Treatment | 0·51 | CBD | Active | 8·43 | 7·09–9·76 |
|  | Phase | 0·92 | Placebo | Active | 7·95 | 6·63–9·28 |
|  | Treatment x Phase | 0·057 | CBD | Inactive | 7·66 | 6·33–8·99 |
|  |  |  | Placebo | Inactive | 8·65 | 7·28–10·01 |

BPI: Brief Pain Inventory short form; CI: Confidence interval; CLMM: Cumulative Link Mixed Model; DASS: Depression Anxiety Stress Scales; DN4: Douleur Neuropathique 4; LSMean: Least Squares Means; NPQ: Neuropathic Pain Questionnaire Short Form; PCS: Pain Catastrophizing Scale; PSQI: Pittsburgh Sleep Quality Index; SE: Standard Error; STAI: State-Trait Anxiety Inventory; SQRT: square root.

### **Table 4** Proportion of participants achieving ≥10%, ≥20%, ≥30%, and ≥50% pain reduction during active treatment.

| **Improvement threshold** | **CBD** | **Placebo** | **Risk Difference (CBD–Placebo)** | **95% CI for Risk Difference** | **p‑value** |
| --- | --- | --- | --- | --- | --- |
| ≥10% | 56.80% | 36.10% | '+20.7% | −4.7% to +43.1% | 0.106 |
| ≥20% | 43.20% | 27.80% | '+15.4% | −9.1% to +37.6% | 0.227 |
| ≥30% | 37.80% | 11.10% | '+26.7% | '+6.0% to +44.0% | **0.014 *** |
| ≥50% | 13.50% | 2.80% | '+10.7% | −4.0% to +23.8% | 0.2 |

CBD: cannabidiol; CI: confidence interval.

### **Figure 1** Daily Pain Intensity During the Cannabidiol (CBD) Treatment Periods defined by 30% responder rate categories. Absolute pain intensity on a 11-point Visual Analogue Scale (VAS).


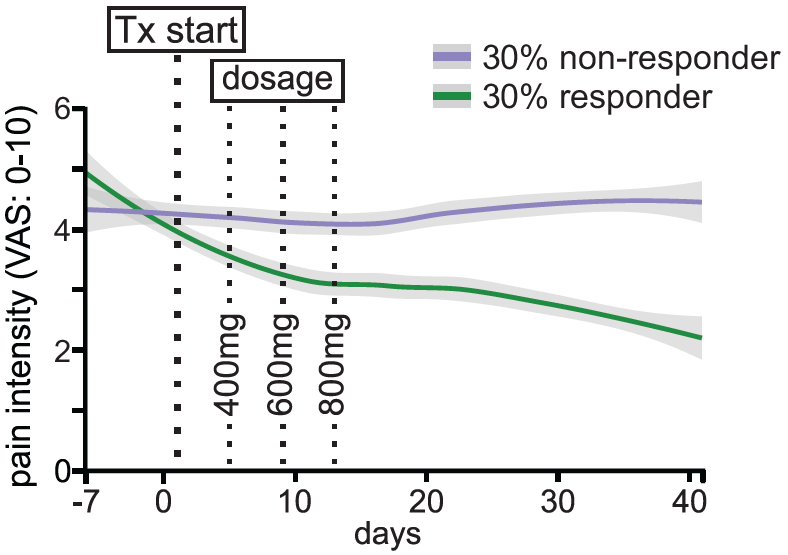


### **Table 5** Association between clinical measures and pain reduction during CBD treatment.

| **Analysis** | **Test** | **Statistic** | **95% CI** | **Effect Size** | **p‑value** |
| --- | --- | --- | --- | --- | --- |
| AIS score | Spearman ρ | 0.1 | −0.24 to 0.44 | — | 0.553 |
| Type of injury | Kruskal–Wallis H | 0.64 | — | η² = 0.018 | 0.425 |
| Time since SCI (years) | Spearman ρ | 0.38 | 0.06 to 0.63 | — | **0.021*** |
| Time since pain onset (years) | Spearman ρ | 0.39 | 0.08 to 0.64 | — | 0.017 |

AIS: American Spinal Injury Association (ASIA) Impairment Scale; CI: confidence interval; SCI: Spinal Cord Injury.

### **Table 6** Association between pain reduction during CBD and plasma analyte measures.

| **Analyte** | **Pearson r** | **95% CI** | **p‑value** | **Regression slope (β)** | **95% CI for β** | **p‑value (β)** | **R²** |
| --- | --- | --- | --- | --- | --- | --- | --- |
| CBD | 0.186 | −0.20 to 0.52 | 0.344 | 0.00047 | −0.00054 to 0.00148 | 0.344 | 0.035 |
| 7‑COOH‑CBD | −0.088 | −0.45 to 0.30 | 0.657 | −1.47×10⁻⁵ | −8.17×10⁻⁵ to 5.24×10⁻⁵ | 0.657 | 0.008 |
| 6‑OH‑CBD | 0.023 | −0.35 to 0.39 | 0.909 | 0.00102 | −0.0172 to 0.0192 | 0.909 | 0.001 |
| 7‑OH‑CBD | 0.123 | −0.26 to 0.47 | 0.532 | 0.00052 | −0.00116 to 0.00219 | 0.532 | 0.015 |

CI: confidence interval.

### **Table 7** Comparison of secondary outcome changes between CBD responders and non‑responders (CBD only).

| **Variable** | **Test used** | **Responder mean** | **Non-responder mean** | **Difference** | **Lower CI** | **Upper CI** | **p-value** |
| --- | --- | --- | --- | --- | --- | --- | --- |
| PCS Rumination | Wilcoxon | -1.33 | -0.235 | -1.1 | -4.24 | 2.04 | 0.337 |
| PCS Magnification | Wilcoxon | -0.333 | -0.0588 | -0.275 | -2.23 | 1.68 | 0.435 |
| PCS Helplessness | Wilcoxon | 0.111 | -0.588 | 0.699 | -2.8 | 4.19 | 1 |
| PCS Score | t-test | -1.56 | -0.882 | -0.673 | -7.46 | 6.11 | 0.826 |
| STAI total | t-test | 0.111 | 2.29 | -2.18 | -13.7 | 9.32 | 0.668 |
| STAI State | t-test | 0.222 | 0.824 | -0.601 | -7.71 | 6.51 | 0.85 |
| STAI Trait | t-test | -0.333 | 1.35 | -1.69 | -5.4 | 2.02 | 0.315 |
| BPI Intensity | t-test | -0.417 | 0.706 | -1.12 | -1.86 | -0.384 | **0.00785 **** |
| BPI Interference | t-test | 0.914 | 1.04 | -0.129 | -2.58 | 2.32 | 0.924 |
| BPI Interference (non-walking) | t-test | 0.296 | 0.833 | -0.537 | -2.16 | 1.08 | 0.51 |
| PSQI Global | t-test | 1.67 | 0.643 | 1.02 | -1.74 | 3.79 | 0.565 |
| DASS Stress | t-test | -0.333 | -1 | 0.667 | -3.17 | 4.51 | 0.718 |
| DASS Anxiety | t-test | -1.44 | -0.294 | -1.15 | -3.77 | 1.47 | 0.418 |
| DASS Depression | Wilcoxon | 1.11 | -0.529 | 1.64 | -2.76 | 6.04 | 0.498 |
| NPQ | t-test | -0.298 | -0.377 | 0.0795 | -0.526 | 0.685 | 0.795 |

BPI: Brief Pain Inventory short form; DASS: Depression Anxiety Stress Scales; DN4: Douleur Neuropathique 4; NPQ: Neuropathic Pain Questionnaire Short Form; PCS: Pain Catastrophizing Scale; PSQI: Pittsburgh Sleep Quality Index; STAI: State-Trait Anxiety Inventory; SQRT: square root.

### **Table 8** Rates of comorbidities between 30% responders and non-responders.

| **Comorbid health conditions** | **No. (%)** | | |
| --- | --- | --- | --- |
|  | **Responder** | **Non-Responder** | **NA** |
| **cardiovascular** | 2 (40) | 3 (60) |  |
| **gastrointestinal** | 2 (67) | 1 (33) |  |
| **musculoskeletal** | 2 (67) | 1 (33) |  |
| **hematopoietic lymphatic** | 2 (67) | 1 (33) |  |
| **endocrine-metabolic** | 0 | 1 (50) | 1 (50) |
| **renal genitourinary** | 0 | 1 (100) |  |
| **respiratory** | 0 | 1 (100) |  |
| **ear, nose, or throat** | 1 (50) | 1 (50) |  |
| **malignancy** | 1 (50) | 1 (50) |  |
| **genetic disorders** | 1 (50) | 1 (50) |  |

* % of individuals who have this comorbid health condition which fall into either responder or non-responder classification.

### **Table 9** Frequency of reported adverse events.

| **Adverse Event** | **CBD (n)** | **Placebo (n)** | **Post CBD Washout (n)** |
| --- | --- | --- | --- |
| **Unusual tiredness/sleepiness** | 15 | 15 | 0 |
| **Nausea** | 10 | 6 | 0 |
| **Feeling unwell** | 7 | 9 | 0 |
| **Diarrhoea** | 7 | 5 | 0 |
| **Loss of appetite** | 6 | 2 | 0 |
| **Pain or discomfort in stomach** | 5 | 4 | 0 |
| **Itching** | 4 | 1 | 0 |
| **UTI** | 3 | 2 | 0 |
| **Rash** | 2 | 0 | 0 |
| **Fever** | 1 | 1 | 0 |
| **Indigestion** | 1 | 1 | 0 |
| **Fractured femur** | 1 | 0 | 0 |
| **Decreased spasms** | 1 | 0 | 0 |
| **Difficulty falling asleep** | 1 | 0 | 0 |
| **Hallucinations** | 1 | 0 | 0 |
| **Increased spasms** | 1 | 2 | 0 |
| **Pneumonia** | 1 | 0 | 0 |
| **Increased muscle stiffness** | 0 | 1 | 0 |
| **Reduced mobility** | 0 | 1 | 0 |
| **Unusually low BP** | 0 | 1 | 0 |
| **Stroke** | 0 | 0 | 1 |

### **Table 10** Most frequently reported adverse events likely related to treatment.

| **Adverse Event** | **CBD (n)** | **Placebo (n)** |
| --- | --- | --- |
| **Unusual tiredness/sleepiness** | 11 | — |
| **Nausea** | 6 | 4 |
| **Diarrhoea** | 3 | 1 |
| **Loss of appetite** | 3 | — |
| **Pain/discomfort in stomach** | 3 | 2 |
| **Itching** | 2 | — |
| **Difficulty falling asleep** | 1 | — |
| **Indigestion** | 1 | 1 |

**NB:** “–” indicates the AE was not reported or not considered likely related in that treatment.

### **Figure 2** Plasma cannabidiol (CBD) concentrations across the trial period, shown pre- and post-treatment for both arms, separated by treatment order (CBD first vs. placebo first). Individual participant trajectories are shown with dotted lines. Values below the limit of detection and lower limit of quantification are represented as 0 ng/mL.


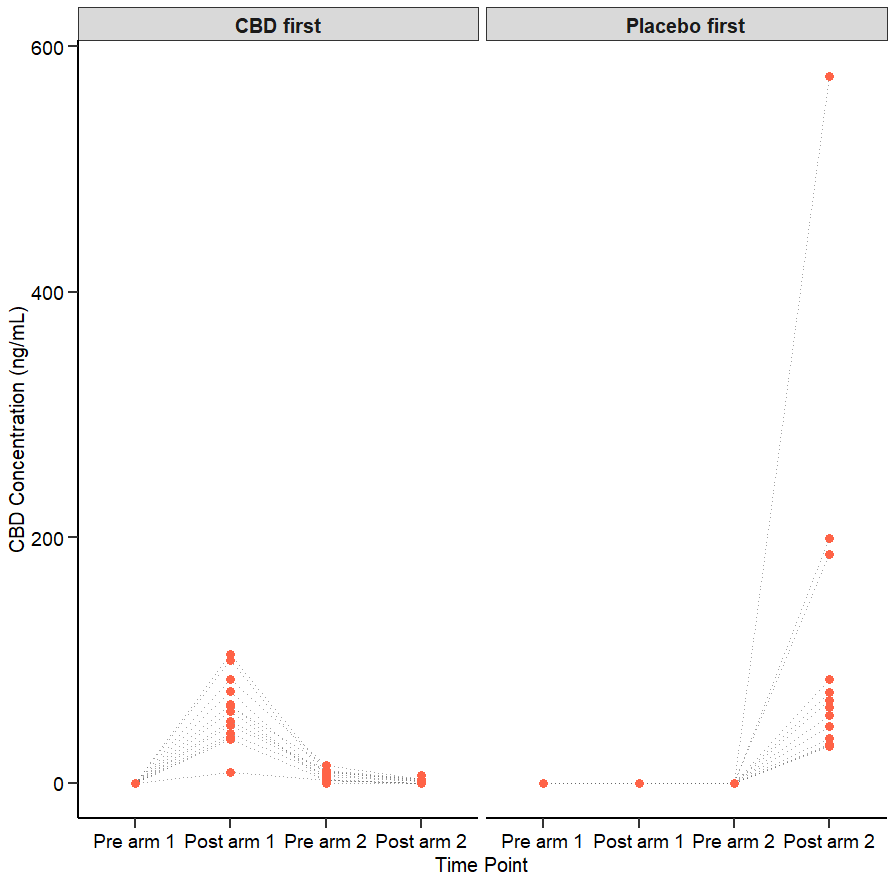


### **Figure 3** Plasma (A) 6 OH CBD, (B) 7 COOH CBD, and (C) 7 OH CBD concentrations across the trial period, shown pre- and post-treatment for both arms, separated by treatment order (CBD first vs. placebo first). Individual participant trajectories are shown with dotted lines. Values below the limit of detection and lower limit of quantification are represented as 0 ng/mL.


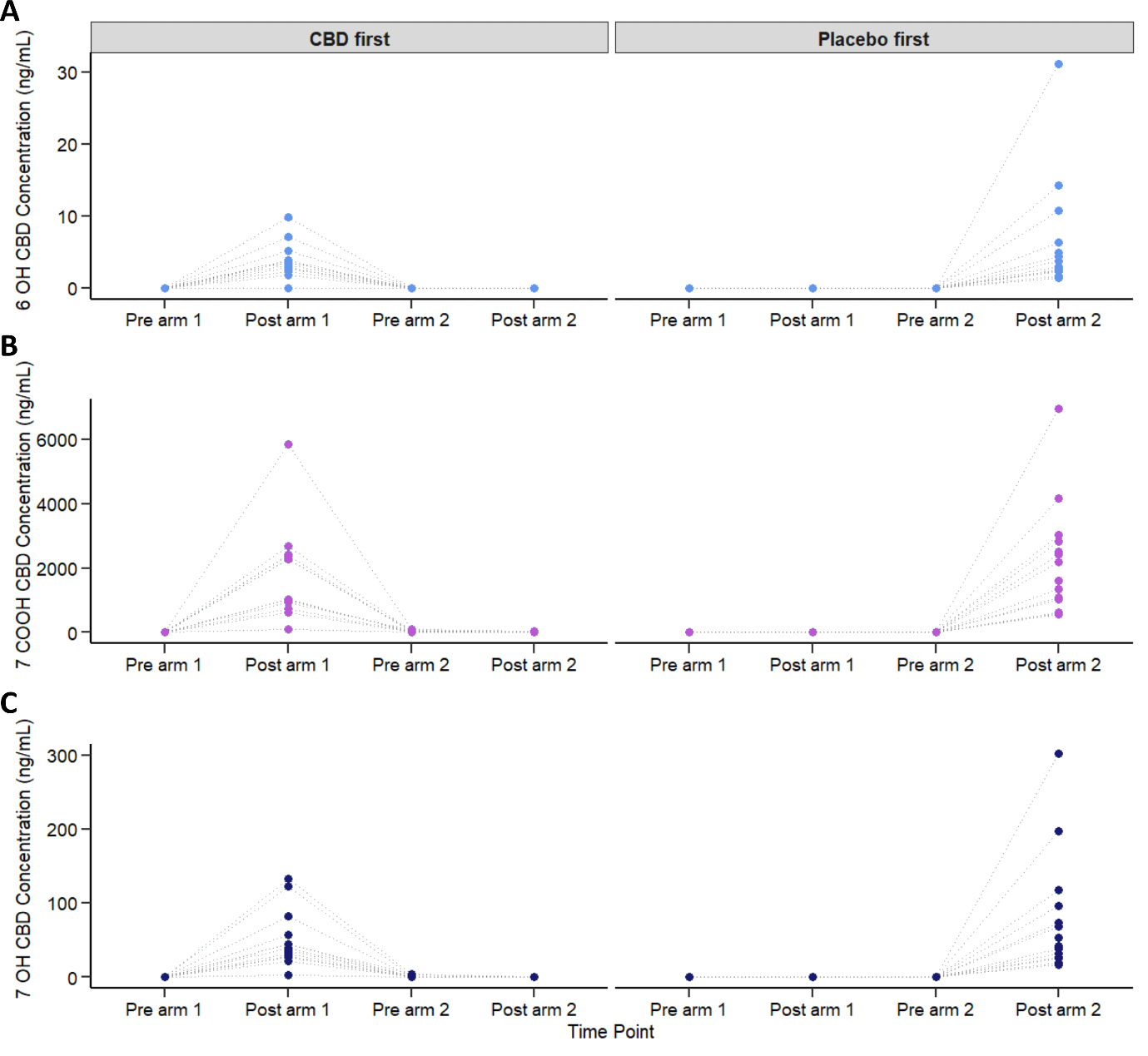


### **Table 11** Expectation, confidence, and accuracy of guess at the end of each treatment phase.

| **Confidence Level** | **Group** | **n (%)** | **Accuracy (%)** |
| --- | --- | --- | --- |
| **Extremely** | **CBD** | 14 (41%) | 43% |
|  | **Placebo** | 16 (48%) | 81% |
| **Moderately** | **CBD** | 14 (41%) | 64% |
|  | **Placebo** | 10 (30%) | 70% |
| **Somewhat** | **CBD** | 4 (12%) | 50% |
|  | **Placebo** | 7 (21%) | 71% |
| **Not at all** | **CBD** | 2 (6%) | 50% |
|  | **Placebo** | 0 (0%) | — |
| — | **Unsure** | 1 | — |

*Note.* Accuracy reflects the percentage of participants whose treatment expectation matched their actual treatment. Percentages for *n* are within-group.

### **Table 12** Intention to seek CBD post-trial and accuracy of guess at the end of each treatment phase.

| **CBD post-trial** | **CBD** | | **Placebo** | | **Unsure**  **(*n*, %)** |
| --- | --- | --- | --- | --- | --- |
|  | **Correct**  **(*n*, %)** | **Incorrect**  **(*n*, %)** | **Correct**  **(*n*, %)** | **Incorrect**  **(*n*, %)** |  |
| **Intend to seek CBD prescription**  (n = 18, 52.9%) | 12 (66.7%) | 6 (33.3%) | 14 (77.8%) | 2 (11.1%) | 1 (5.6%) |
| **Do not intend**  (n = 13, 38.2%) | 4 (30.8%) | 9 (69.2%) | 8 (61.5%) | 3 (23.1%) | 2 (15.4%) |
| **Unsure**  (n = 3, 8.8%) | 2 (66.7%) | 1 (33.3%) | 3 (100%) | 0 (0%) | 0 (0%) |

### **Table 13** Association of FAAH variants with ≥30% pain reduction after CBD and placebo treatment.

| **SNPs** | **Treatment** | **Genotype** | **Responders / Total (%)** | **Odds Ratio (95% CI)** | ***P* value** |
| --- | --- | --- | --- | --- | --- |
| rs324420 | CBD | A-allele carrier | 8/16^ (50.0%) | 2.00 (0.48–9.03) | 0.35 |
|  |  | Non allele carrier | 6 / 15^ (40.0%) | Ref | — |
|  | Placebo | A-allele carrier | 2 / 16^ (12.5%) | 2.00 (0.17–45.99) | 0.59 |
|  |  | Non allele carrier | 1 / 15^ (6.7%) | Ref | — |
| rs3766246 | CBD | A-allele carrier | 12 / 22 (54.6%) | 3.50 (0.66–27.20) | 0.17 |
|  |  | Non allele carrier | 2 / 9 (22.2%) | Ref | — |
|  | Placebo | A-allele carrier | 3 / 23 (13.0%) | Model unstable^&^ | — |
|  |  | Non allele carrier | 0 / 8 (0.0%) | — | — |

* Wide confidence intervals reflect small sample sizes within some genotype groups (≤5 participants).

^^^ Responders vs. non-responders

^&^ Logistic regression model estimates were unstable due to quasi-complete separation; odds ratio and confidence intervals could not be reliably estimated.

Ref., Reference group.

### **Research Protocol and Statistical Plan**

Please double click image to open:
